# Alteration of circulating ACE2-network related microRNAs in patients with COVID-19

**DOI:** 10.1038/s41598-024-58037-3

**Published:** 2024-06-12

**Authors:** Zofia Wicik, Ceren Eyileten, Anna Nowak, Disha Keshwani, Sérgio N. Simões, David C. Martins, Krzysztof Klos, Wojciech Wlodarczyk, Alice Assinger, Dariusz Soldacki, Andrzej Chcialowski, Jolanta M. Siller-Matula, Marek Postula

**Affiliations:** 1 Department of Experimental and Clinical Pharmacology, Center for Preclinical Research and Technology CEPT, Medical University of Warsaw, 02-097 Warsaw, Poland; 2 Department of Neurochemistry, Institute of Psychiatry and Neurology, Sobieskiego 9 Street, 02-957 Warsaw, Poland; 3 Genomics Core Facility, Centre of New Technologies, University of Warsaw, Warsaw, Poland; 4 Doctoral School, Medical University of Warsaw, 02-091 Warsaw, Poland; 5 Department of Diabetology and Internal Medicine, University Clinical Centre, Medical University of Warsaw, Warsaw, Poland; 6 Federal Institute of Education, Science and Technology of Espírito Santo, Serra, Espírito Santo 29056-264 Brazil; 7 Centro de Matemática, Computação e Cognição, Universidade Federal do ABC, Santo Andre, 09606-045 Brazil; 8 Department of Infectious Diseases and Allergology - Military Institute of Medicine, Warsaw, Poland; 9 Department of Vascular Biology and Thrombosis Research, Center of Physiology and Pharmacology, Medical University of Vienna, Vienna, Austria; 10 Department of Clinical Immunology, Medical University of Warsaw, Warsaw, Poland; 11 Department of Internal Medicine II, Division of Cardiology, Medical University of Vienna, 1090 Vienna, Austria

**Keywords:** Data mining, Machine learning, Predictive medicine, Biomarkers, Cardiology, Diseases, Medical research

## Abstract

Angiotensin converting enzyme 2 (ACE2) serves as the primary receptor for the SARS-CoV-2 virus and has implications for the functioning of the cardiovascular system. Based on our previously published bioinformatic analysis, in this study we aimed to analyze the diagnostic and predictive utility of miRNAs (miR-10b-5p, miR-124-3p, miR-200b-3p, miR-26b-5p, miR-302c-5p) identified as top regulators of ACE2 network with potential to affect cardiomyocytes and cardiovascular system in patients with COVID-19. The expression of miRNAs was determined through qRT-PCR in a cohort of 79 hospitalized COVID-19 patients as well as 32 healthy volunteers. Blood samples and clinical data of COVID-19 patients were collected at admission, 7-days and 21-days after admission. We also performed SHAP analysis of clinical data and miRNAs target predictions and advanced enrichment analyses. Low expression of miR-200b-3p at the seventh day of admission is indicative of predictive value in determining the length of hospital stay and/or the likelihood of mortality, as shown in ROC curve analysis with an AUC of 0.730 and a p-value of 0.002. MiR-26b-5p expression levels in COVID-19 patients were lower at the baseline, 7 and 21-days of admission compared to the healthy controls (P < 0.0001). Similarly, miR-10b-5p expression levels were lower at the baseline and 21-days post admission (P = 0.001). The opposite situation was observed in miR-124-3p and miR-302c-5p. Enrichment analysis showed influence of analyzed miRNAs on IL-2 signaling pathway and multiple cardiovascular diseases through COVID-19-related targets. Moreover, the COVID-19-related genes regulated by miR-200b-3p were linked to T cell protein tyrosine phosphatase and the HIF-1 transcriptional activity in hypoxia. Analysis focused on COVID-19 associated genes showed that all analyzed miRNAs are strongly affecting disease pathways related to CVDs which could be explained by their strong interaction with the ACE2 network.

## Introduction

Coronavirus disease 2019 (COVID-19) is caused by respiratory tract infection with severe acute respiratory syndrome coronavirus 2 (SARS-CoV-2)^1,2^. It was found that the SARS-CoV-2 virus enters cells through angiotensin converting enzyme 2 (ACE2) cell membrane enzymes, simultaneously leading to the downregulation in the ACE2-related pathways. This enzyme is abundantly present in the lungs, heart and endothelium of arteries and veins, among others and is involved in the pathogenesis of hypertension, heart failure (HF), diabetes mellitus (DM) and lung diseases. Thus, the impairment of ACE2 functions may result in myocardial injury, fibrosis, and inflammation, and, what follows, adverse cardiac outcomes^3,4^.

In line with these, SARS-CoV-2 infections and related acute respiratory distress syndrome (ARDS), were found to be alongside the occurrence of myocardial damage and HF acute kidney injury, coagulopathy, and arrhythmias. The occurrence of myocardial injury varied between 7 to 28% based on the severity of COVID-19. It was accompanied by increased levels of well-known biomarkers of myocardial damage—cardiac troponins, creatine kinase–myocardial band, myo-hemoglobin, and N-terminal pro-B-type natriuretic peptide (NT-proBNP)^5^. However, the exact pathogenesis of COVID-19 related cardiovascular complications remains unknown.

Classification of clinical data, also COVID-19 related as early as possible, is an important goal to speed up the diagnostic process and support efficient decision-making^6,7^. Since time is an essential factor in the hospitalization process, a slight delay in decision-making could generate costs and risks for patient health. Using Machine Learning (ML) models allows to make analysis more time efficient in predictive performance. Thus, alongside traditional statistical tools in our study, we used SHapley Additive exPlanations (SHAP) to increase interpretability of our clinical dataset and identify features associated with death and long-term hospitalization. SHAP tool was developed for making ML models more explainable^8^. Shapley values are a solution concept from game theory and it provides weights or relations describing how impactful are different features playing in determining the output of the model^9^. Supervised ML methods like SHAP, involving labeled data, can capture complex interactions and non-linear associations among explanatory variables. In this case we used this information to evaluate the functions of miRNAs associated with COVID-19 hospitalization length.

MicroRNAs (miRNAs) are a class of small, endogenous non-coding RNAs, (ncRNAs) ncRNAs, that play an important role in a wide range of biological processes. MiRNAs interact with the 3′ untranslated region (3′ UTR) of target mRNAs which stimulates mRNA degradation and translational repression^10^. For example plasma miR-195-5p predicts the severity of Covid-19 in hospitalized patients. Authors showed that plasma miR-195 correlates with several clinical and paraclinical parameters, and is an excellent discriminator between the severe and mild forms of the disease^11^. In other work some miRNAs, including miR-24-3p, whose differential expression was discovered in patients with acute lung injury complications brought on by severe COVID-19, and miR-148a-3p, differentially expressed against SARS-CoV-2 structural proteins, were identified, thereby suggesting the effectiveness and accuracy of they machine learning framework^12^. In other study focused on miRNome analysis was constructed a mortality predictive risk score (miRNA-MRS) with ten miRNAs. Patients with higher values had a higher risk of 90-days mortality (hazard ratio = 4.60; p-value < 0.001). Besides, the discriminant power of miRNA-MRS was significantly higher than the observed age and gender (AUROC = 0.970 vs. 0.881; p = 0.042)^13^. Also in our previous study focused on coagulation related complications in COVID, by using bioinformatic predictions we identified and validated that miR-16-5p and let-7b in COVID-19 patients were lower at baseline, 7-days and 21-day after admission compared to the healthy controls (p < 0.0001 for all time points for both miRNAs). The expression levels of miR-27a-3p and miR-155-5p in COVID-19 patients were higher at day 21 compared to the healthy controls (p = 0.007 and p < 0.001, respectively)^2^. Those several miRNAs and their targets, which are involved in regulation of ACE2 interaction networks that can oblige as potential novel biomarkers for identification of the pathological alterations in COVID-19 or serve as therapeutic targets^1,14–16^. We believe that current availability of literature strongly supports the usefulness of miRNAs as predictors of COVID progression.

Consequently, based on our previously published bioinformatic analysis, in this study we aimed to analyze the diagnostic and predictive utility of miRNAs (miR-10b-5p, miR-124-3p, miR-200b-3p, miR-26b-5p, miR-302c-5p) identified as top regulators of ACE2 network with potential to affect cardiomyocytes and cardiovascular system in patients with COVID-19.

## Results

### Participants

No differences were observed between healthy individuals and COVID-19 patients in regards to basic demographic data including age, sex, and body mass index (BMI) (P = 0.254, P = 0.707, P = 0.421, respectively). From the 79 patients, nine of them (11.4%) admitted to intensive care units (ICU), died within the median of 11 days, as published before^2^. Cardiovascular diseases of analyzed patients included myocardial infarction, coronary artery disease and atrial fibrillation. We present patient characteristics in Tables 1 and 2.

**Table 1:** Participant’s characteristics.

Participant’s characteristics				
Characteristic	COVID-19 patients (N = 79)	Healthy individuals (N = 32)		*P*-value
Sex (male)	44 (55.7%)	14 (44%)		0.254
Age	59.7 ± 14.6	58.47 ± 16.59		0.707
BMI	29.7 ± 6.58	28.6 ± 6.3		0.421
Hypertension	35 (44.3%)	16 (50%)		0.585
DM	17 (21.5%)	6 (17%)		0.525
Current smoking	5 (6.3%)	4 (10%)		0.543
Asthma/COPD	6 (7.6%)	3 (6.3%)		0.756
CVD (CAD, MI, AF)	14 (17.7%)	4 (12.5%)		0.499

Patients characteristics				
Laboratory parameters	At admission (N = 79)	Day-7 (N = 71)	Day-21 (N = 18)	*P*-value
CRP	5.3 [3.13–769]	1.87 [0.61–5.27]	0.5 [0.1–4.89]	** < 0.001**
D-dimer	1.15 [0.45–2.16]	0.80 [0.46–1.47]	0.72 [0.36–1.78]	0.654
PCT	0.11 [0.07–0.14]	0.07 [0.04–0.09]	0.07 [0.04–0.13]	0.538
WBC	6.49 ± 2.87	8.69 ± 2.81	6.98 ± 2.74	**0.028**
Neutrophils	4.58 ± 3.17	6.24 ± 2.75	4.23 ± 2.0	0.091
Lymphocyte	0.72 ± 0.28	1.46 ± 0.85	1.74 ± 0.97	**0.015**
Ferrum	36.5 [26.25—59.25]	86 [72.75—106.75]	88.5 [51.25—115.5]	**0.001**
Ferritine	633 ± 306.20	771 ± 569.76	855 ± 310.23	0.093

The data is presented in two ways, encompassing both the mean ± standard deviation (SD) and the median with the interquartile range, which provide insights into the characteristics of the data distribution. The P-value is calculated by employing a One-way ANOVA test for the purpose of comparing three groups. Abbreviations used include: AF for Atrial fibrillation, CAD for coronary artery disease, MI for myocardial infarction, BMI for body mass index, COPD for chronic obstructive pulmonary disease, CRP for C-reactive protein, CVD for cardiovascular disease, DM for diabetes mellitus, PCT for procalcitonin, SD for standard deviation, and WBC for white blood cells.

Significant values are in bold.

**Table 2:** A multivariable logistic regression model was employed to predict extended hospitalization and/or mortality, incorporating the delta of miR-200b-3p alongside clinical variables.

Variable	OR	95% CI		p-value
		Lower	Upper	
Low expression of delta miR-200b-3p	5.775	1.572	21.214	**0.008**
Gender (male)	0.452	0.105	1.940	0.286
Age	1.001	0.940	1.066	0.979
BMI > 30	1.954	0.493	7.749	0.341
DM	4.888	1.001	23.858	**0.050**
Smoking	0.823	0.044	15.446	0.896
Hypertension	0.797	0.155	4.092	0.786
CVD	0.390	0.064	2.365	0.306

Significant values are in bold.

*OR* odd ratio, *CI* confidence interval, *miR* microRNA, *BMI* body mass index, *CVD* cardiovascular disease.

Patients’ characteristics at 3 different time-points (at hospital admission, day-7 and day-21 during hospitalization) are shown in Table 2. Significant differences were observed between the timepoints in regards to the WBC count (P = 0.028). Also the increase in lymphocyte count and ferrum levels (P = 0.015, P = 0.001 respectively), together with the reduction in CRP levels (P < 0.001) were noted between the timepoints.

### Selection of the analyzed miRNAs

**Figure 1:**
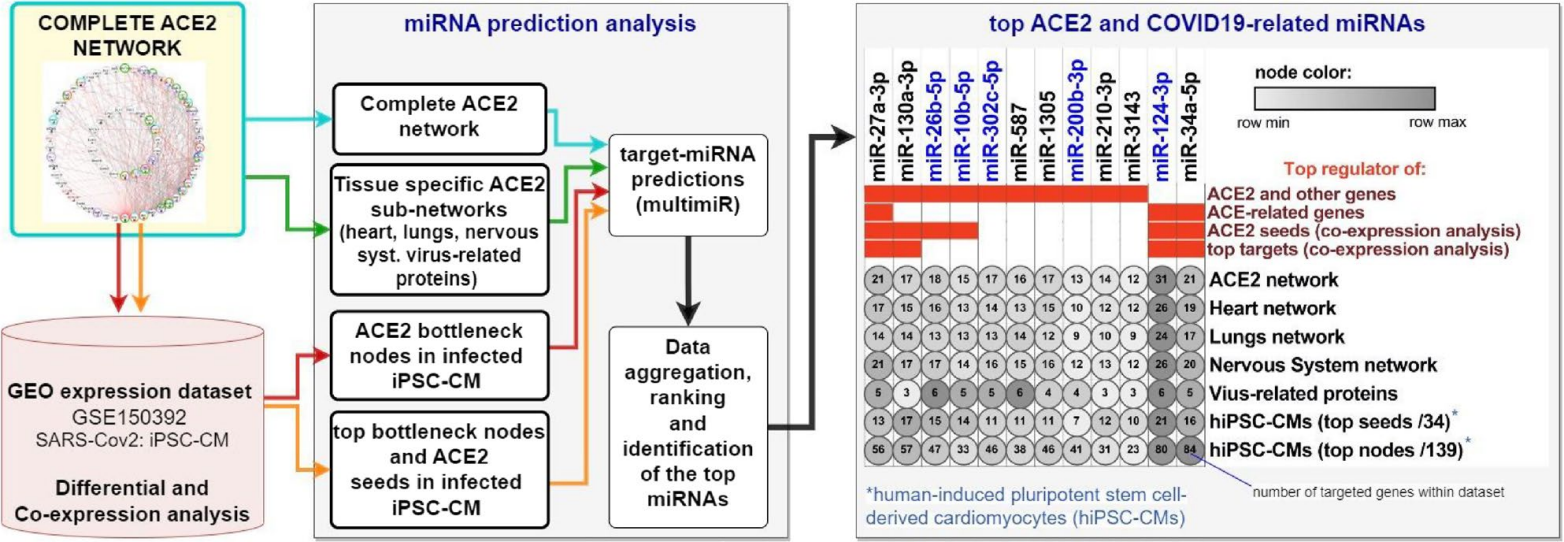
Top predicted miRNA modulators of the ACE2 network in COVID-19 and workflow leading to their identification. Statistics for top miRNAs regulating the highest number of genes within ACE2 related-networks based on previous in silico predictions^1^. Red squares show if miRNA was present among the top miRNAs in a analyzed dataset. MiRNAs validated in present study using qRT-PCR have blue labels.

According to our previously published bioinformatics analysis, we aimed to analyze the diagnostic and predictive utility of miRNAs (miR-10b-5p, miR-124-3p, miR-200b-3p, miR-26b-5p, miR-302c-5p) identified as top regulators of ACE2 network with potential to affect cardiomyocytes and cardiovascular system in patients with COVID-19^1^. Additionally using bioinformatic tools we predicted their targets and associated ontological terms which could play a role in COVID-19 progression. Selection workflow of the analyzed miRNAs is demonstrated in Fig. 1.

### Evaluation of the alterations in circulating ACE2-regulating miRNAs

**Figure 2:**
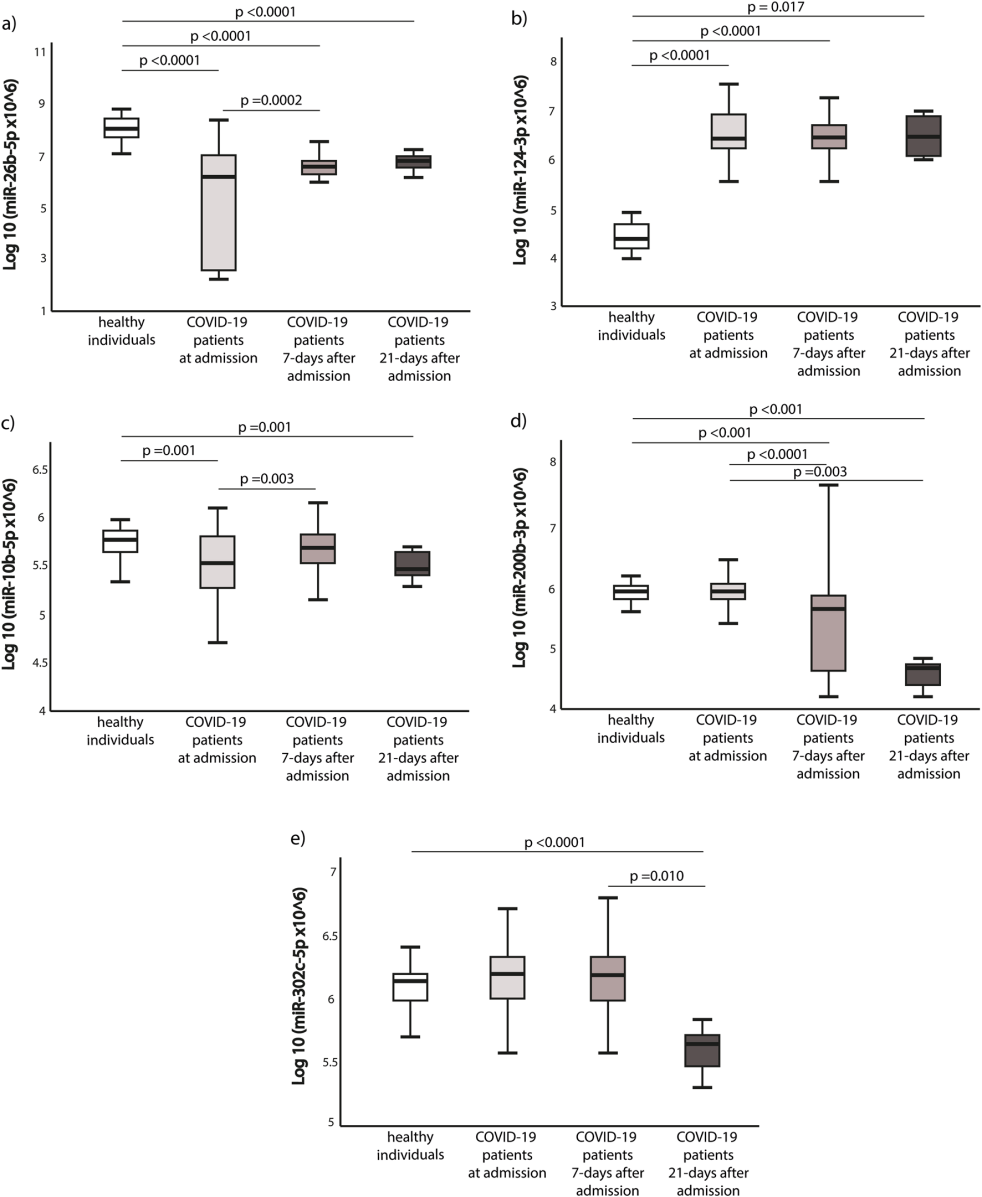
Comparison of ACE2 and COVID19 related miRNAs expression in different groups. The Wilcoxon and Mann–Whitney U test and test were appropriately employed, while the Kruskal–Wallis test was utilized to detect differences among the four groups. The signal was obtained for each miRNA from the following number of samples: (**a**) miR-26b-5p (81.01%), (**b**) miR-124-3p (46.83%), (**c**) miR-10b-5p (93.67%), (**d**) miR-200b-3p (94.94%), (**e**) miR-302c-5p (93.67%). The miRNA expression data is presented after applying logarithmic transformation correction. Lower delta expression of miR-200b-3p predicts increased hospital length of stay and/or death. Results of Kruskal–Wallis test for all comparisons had p < 0.001, except (**c**) where it was p = 0.001.

Assessment of changes in ACE2-regulating miRNAs is depicted in Fig. 2. It illustrates the relative expression of circulating miRNAs in healthy individuals versus COVID-19 patients, at three different time intervals: upon admission, 7 days post-admission, and 21 days post-admission. Our research uncovered that, in COVID-19 patients, miR-26b-5p expression levels were notably reduced at baseline, 7 days post-admission, and 21 days post-admission when compared to those of healthy controls (p < 0.0001 at all-time points). Similarly, miR-10b-5p displayed reduced expression levels in COVID-19 patients compared to healthy individuals at both the baseline and 21 days after admission (p = 0.001 at both time points). Moreover, the expression of this miRNA demonstrated a notable increase 7 days after admission in comparison to the baseline, for which p-value was 0.003. Additionally, there was an observed downward trend in the expression levels of miR-200b-3p among COVID-19 patients. Specifically, it was significantly lower at 7 and 21 days after admission when compared to the baseline expression (p < 0.0001 and p = 0.003, respectively) and when contrasted with healthy individuals (p < 0.001 at both time points). The opposite situation was observed in miR-124-3p and miR-302c-5p concentrations. MiR-124-3p expression was significantly higher at the baseline, 7-days and 21-days post-admission when compared to the control group (P < 0.001, P < 0.001 and P = 0.017 respectively). In miR-302c-5p the significant differences were shown in the decrease of its expression in patients 21-days post-admission when compared to the control group (P < 0.0001) and patients 7-days after admission (P = 0.010).

**Figure 3:**
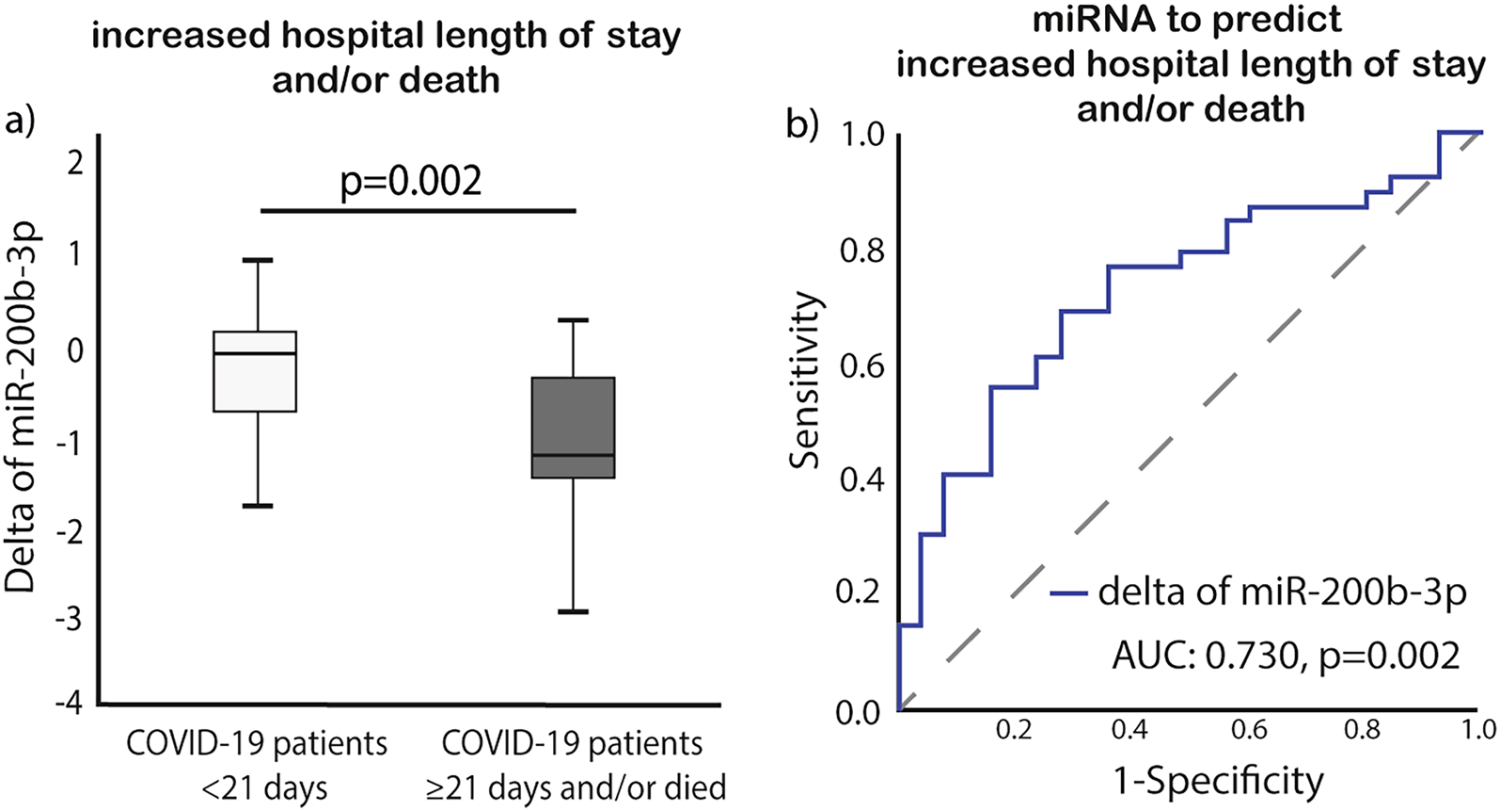
Delta of miR-200b-3p (7 days after admission compared to the day of admission) (**a**) box-plots comparing increased hospital length of stay and/or mortality, and (**b**) ROC curves for predicting hospital length of stay and/or death. Abbreviations used: AUC for Area under the ROC Curve, COVID-19 for coronavirus disease 2019, and miR for microRNA.

We found that MiR-200b-3p expression was significantly and negatively correlated with the hospital length of stay and/or death. Patients who were hospitalized over 21 days and/or died had significantly lower expression levels of miR-200b-3p when compared to those hospitalized under 21 days (P = 0.002). The ROC curve analysis demonstrated that reduced delta miR-200b-3p expression has predictive value in evaluating both the length of hospital stay and the likelihood of mortality, with an AUC of 0.730 and a p-value of 0.002 (Fig. 3b).

The COVID-19 patient group was stratified into two subgroups using ROC curve analysis, considering changes in miR-200b-3p expression (delta), specifically, distinguishing between low and high values. This stratification was based on factors such as extended hospitalization or the occurrence of mortality (Fig. 3a).

The threshold of -0.522 was set to define a low delta miR-200b-3p expression level, encompassing 44% of the population. The area under the curve (95% CI) was 0.730 (0.61–0.86), with a positive predictive value (PPV) of 60% and a negative predictive value (NPV) of 79%. The sensitivity and specificity for this threshold were 69% and 72%, respectively (Supplemental Table 1).

As per the multivariable logistic regression model, both low delta miR-200p-3p expression and the presence of diabetes mellitus (DM) independently predict extended hospitalization and/or mortality (odds ratio: 5.775; 95% confidence interval, 1.572–21.214; p = 0.008 and odds ratio: 4.888; 95% confidence interval, 1.001–23.858; p = 0.050, respectively) (Table 2).

### Results of machine learning based analysis

**Figure 4:**
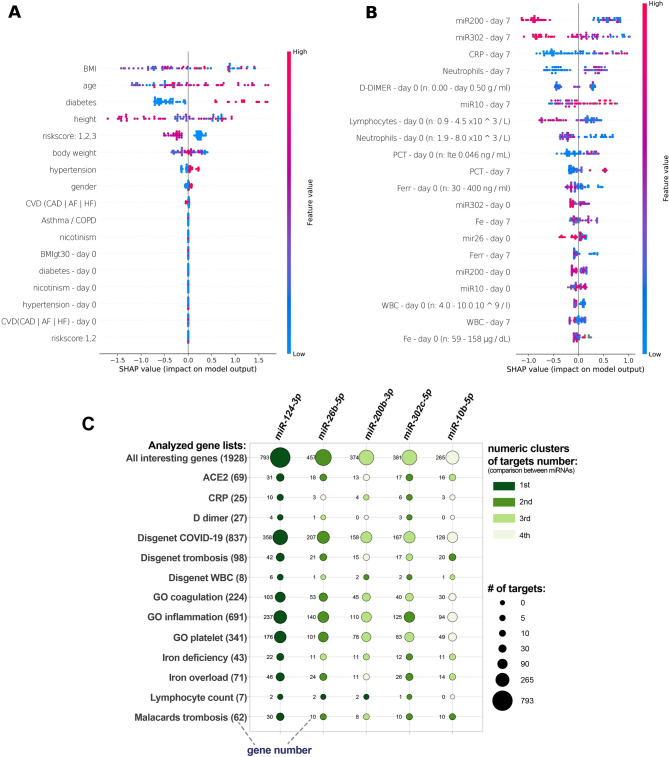
Results of SHAP method applied by using XGBoost classifier to the panel (**A**) demographic and panel (**B**) laboratory part of COVID19 data. Results show the contribution locally per sample of each feature to the prediction and the most important features associated with the outcome death + 21 day stay. The features with highest importance for a given outcome are localized on the top of the graph. We highlight the features BMI (from very high and very low spectrum), age (high) and diabetes (positive) from the Demographics section are associated with death + 21 day stay outcome (Fig. 3a). Panel (**C**) Numbers of targets of interest regulated by analysed miRNAs. Analysis was performed separately for each miRNA and then combined. Genes from specific gene lists were divided into four clusters based on their amount using the OneR algorithm.

In order to identify features strongly associated with COVID19 death and long hospitalisation we performed SHAP analysis which calculated the contribution of each feature to the prediction indicating their importance. The SHAP values we acquired enabled us to understand how having a particular value for a specific feature influences the prediction, as opposed to what the prediction would be with a baseline value for that feature. This method helps with the interpretability problem and can be used to improve the classification models (Fig. 4A and B).From the Laboratory section miR-200b-3p on day 7th (low expression), miR-302c-5p on day 7th (low and moderate expression), and CRP on day 7th (high level), neutrophils on day 7th (high and average), D-Dimer on day 0 (very low and very high) appeared in the top important features associated with death + 21daygstay outcome according to SHAP analysis (Fig. 4B). Actually, considering all the executions, miRNA’s miR-200b-3p, miR-302c-5p and miR10b-5p were always present among the top features, which reinforces their importances in predicting the *death* + *21 day stay* outcome. Moreover, while most miR's have high values associated with not death, miR10b-5p on day 0 and miR-10 on day 7th specifically tend to present high values associated with death and long term hospitalization. Additional hierarchical analysis of SHAP values allowed us to identify associations between most important features. We observed redundancy between levels of D-dimer (day 7th) and miR-26-5p (day 0). In summary this machine learning based analysis identified miR-200b-3p levels on the 7th day, miR-302c-5p levels on the 7th day, CRP levels on the 7th day, neutrophil levels on the day of admission, and D-Dimer levels on the day of admission as the most reliable predictors of extended hospitalization in COVID-19 patients.

### Target predictions of analyzed miRNAs in context of clinical data

To clarify the role of analyzed miRNAs in COVID19 hospitalization we performed target predictions in the context of several lists of genes associated with important features identified in clinical data, and processes recognized as playing a role in COVID19 disease, especially related to thrombosis. Prioritization based on the number of regulated targets within each of the analyzed gene lists highlighted miR-124-3p and miR-26b-5p as having an especially high number of targets of our interest. All analyzed miRNAs, except for miR-124-3p were identified as targeting ACE2 directly, besides other components of the ACE2 network. Three miRNAs, miR-124-3p, miR-26b-5p and miR-302c-5p were found targeting D-dimer related genes. The summary results of this analysis are shown in Fig. 4C. A complete list of genes from each list targeted by analyzed miRNAs is available in Supplementary File 2.

### miRNA targets enrichment analysis

Enrichment analysis is a computational method allowing us to identify which biological terms affected are by analysed miRNAs, based on all genes they are targeting. We screened multiple databases provided by EnrichR tool, in order to identify top signalling pathways (Bioplanet_2019), and phenotypes (GWAS_Catalog_2019), affected by ACE2-related miRNAs.

**Pathway enrichment** analysis showed distinct regulation between analysed miRNAs (Supplemental Fig. 1A). Among top shared pathways was BDNF signaling including miR-10b-5p, miR-124-3p, miR-200b-3p. Interleukin-2 signaling pathway, TGF-beta regulation of extracellular matrix and Pathways in cancer were overrepresented for targets of all analysed miRNAs. miR-200b-3p showed regulation of targets associated with developmental pathways, including signaling events mediated by HGFR, Insulin signaling, BDNF pathway and EGF/EGFR signaling.

**GWAS enrichment** analysis for targets of analysed miRNAs, showed multiple significantly enriched terms related to CVD but only for miR-200b-3p and miR-302c-5p (Supplemental Fig. 1B). miR-200b-3p showed association with CVD-related sodium levels, systolic blood pressure and atrial fibrillation. While miR-302c-5p targeted genes associated with systolic blood pressure and QRS duration.

**Figure 5:**
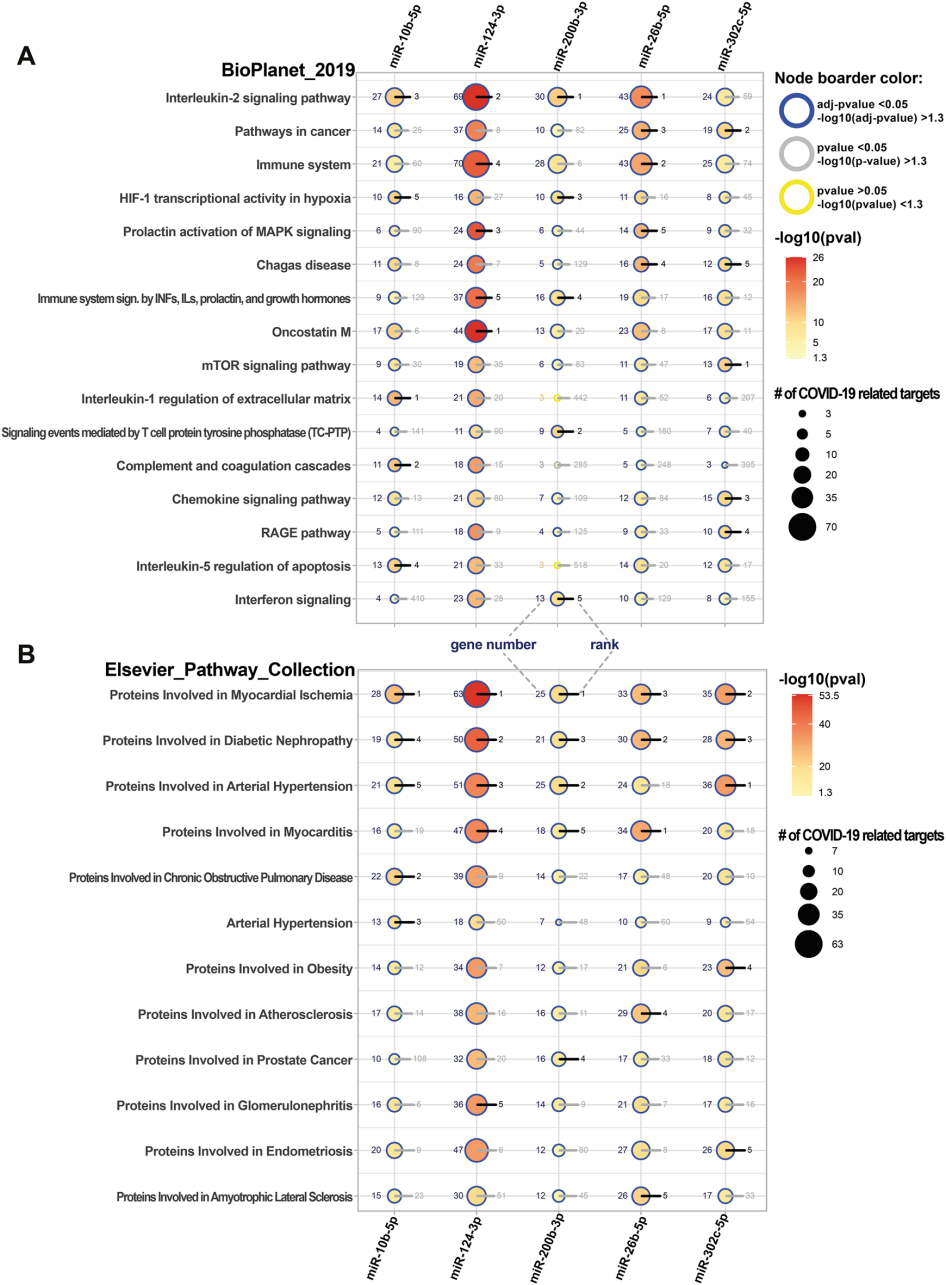
Top 5 significantly enriched signalling pathways (**A**) and disease-related pathways (**B**) associated specifically with COVID19-related targets of ACE2 network related miRNAs. The adjusted p-values (blue boarders) are representing categories which are more likely to have biological meanings. The color gradient is related to corresponding adjusted p-values. Blue color indicates high p-values (low enrichment), and red color indicates low p-values (high enrichment). The dot's size is related to the number of enriched genes. COVID19-related targets were identified using the DisGeNet database.

In order to evaluate which signalling pathways and disease-related pathways can be directly affected during COVID19 by our miRNAs of interest, we first subset COVID19 related targets of those miRNAs. Those targets were mentioned in at least 2 publications according to the DisGeNet database https://www.disgenet.org/covid/diseases/summary/ (837 genes in total). Further on selected targets we performed enrichment analysis using Bioplanet 2019 database and Elsevier pathway collection. Signalling pathway analysis pointed out again IL-2 signalling, but didn’t include BDNF-related signaling (Fig. 5A). Other especially interesting affected pathways included Complement and coagulation cascades for miR-10b-5p; oncostatin M for miR-124-3p; signaling events mediated by T cell protein tyrosine phosphatase (TC-PTP) and HIF-1 transcriptional activity in hypoxia for miR-200b-3p; immune system for miR-26b-5p; and mTOR signaling for miR-302c-5p. Disease related ontological analysis showed that all analysed miRNAs are strongly targeting terms associated with CVDs (Fig. 5B). The most significantly enriched were Proteins Involved in Myocardial Ischemia (miR-200b-3p, miR-124-3p, miR-10b-5p), Proteins Involved in Myocarditis (miR-26b-5p), and Proteins Involved in Arterial Hypertension (miR-302c-5p). Additionally, for miR-200b-3p we observed enrichment of Proteins Involved in Prostate Cancer.

## Discussion

Our previous bioinformatic analysis revealed the ability of **miR-200b-3p** to regulate ACE2 interaction network and differentially expressed genes in heart and Human-induced pluripotent stem cell-derived cardiomyocytes (hiPSC-CMs) infected with SARS-COV-2^1^. Another in silico analysis has shown miR-200b targeting host cell receptor ACE2 and was predicted to be highly expressed in respiratory epithelial cells, which indicates its role in COVID-19 infection^17^. Moreover, lower miR-200 expression is correlated with a lower survival rate in severe and critical clinical conditions^18^. Corresponding with that, in our study group, miR-200b levels were significantly lower in the COVID-19 patient group. Interestingly, the miR-200b-3p transfection alleviated the pulmonary fibrosis resulting in regeneration and slowdown of senescence through restoration of damaged alveolar cells^19^. Our enrichment analysis aligned with those results showing that an important target of this miRNA, are HGFR, EGFR and BDNF pathways associated with fibrosis. Analysis focused only on COVID-19-related targets identified signalling events mediated by TC-PTP and HIF-1 transcriptional activity in hypoxia as regulated by miR-200b-3p. Protein tyrosine phosphatases PTP-1B and TC-PTP are known to play nonredundant roles in macrophage development and IFN-γ signaling and JAK/STAT pathway^20^. Also HIF-1α is a key activator for both SARS-CoV-2 infection and inflammatory response and plays a role in aggravation of COVID19^21^. Those results strongly support the importance of miR-200b-3p as a biomarker and an important player in COVID19 progression.

In previous studies, the role of **miR-26b** was evaluated only by in silico analysis in the context of SARS-CoV2 infection. All three bioinformatic analyses showed miR-26b as a promising monitoring biomarker in COVID-19 and predicted the ACE2 as a strong miR-26b target^22^. Virus S-protein, ACE along with histone deacetylase (HDAC) pathway which are known to have a core role in SARS-CoV2 cellular entry and pathogenicity were mentioned to modulate miR-26b-5p expression levels. Further evaluation of top pathways targeted by miR-26b-5p occurred to be also highly linked and modulated by HDAC. This indicates a particular cross-talk between miR-26b-5p and HDAC that can be used as a therapeutic strategy^23^. Additionally, our previous publication emphasized miR-26b-5p potential role in COVID-19 associated heart failure (HF) progression^1^. In this current study for the first time, we confirmed the bioinformatic analysis in the samples from COVID-19 patients which showed a significant downregulation trend over time. Utilization of Machine learning related SHAP tool and hierarchical clustering allowed us to identify association of hsa-miR-26b-5p level on the first day of hospitalization with the later level of D-dimer (7th day of hospitalization). Target prediction analysis identified that this miRNA targets F3 which according to GWAS studies is associated with D-dimer levels^24^. D-dimer value on admission is a well-recognized and accurate biomarker for predicting mortality in patients with COVID-19^25^. In our study enrichment analysis of COVID-19 related targets of miR-26b pointed out strong regulation of proteins involved in myocarditis. Which is also related to SARS-CoV-2 infection^26^. Literature data shows multiple associations of miR-26b with MI and other CVDs, but so far there are no direct links with myocarditis^27,28^.

**MiR-124-3p** influences a variety of processes including neural functional recovery, adipogenic differentiation and wound healing. In the central nervous system (CNS) miR-124-3p was shown to promote functional repair in animal models of neurodegenerative diseases^29^. However, the role of miR-124 in SARS-CoV2 infection is highly limited. In the human study, the upregulation of miR-1-3p/miR-124-3p ratio was observed among patients who died due to SARS-CoV2 infection, but considering the number of days under invasive mechanical ventilation, the correlation was inverted^30^. Importantly, the latest study aimed to analyze the difference of miR-124 expression in the 5 different groups of COVID-19 patients and showed that the expression of miR-124 in the critically ill group was the highest compared to all the other groups^31^. Our results are in line with this observation as we have found significantly higher miR-124 expressions in the patients in three different time points compared to controls. Our enrichment analysis pointed out myocardial ischemia for its COVID-19 related targets. Literature data are consistent with this finding showing that miR-124 targets various pathways including SIRT1 and STAT3 which promotes ischemia–reperfusion induced cardiomyocyte apoptosis^32^. Additionally, enrichment analysis pointed out oncostatin M as the 1st rank signalling pathway for miR-124-3p. Oncostatin M is a member of the IL-6 family and stands as a mediator in various CVDs such as myocardial infarction or myocarditis and regulates cardiomyocyte remodeling^33^. Additionally for miR-124-3p, miR-302c-5p and miR-26b-3p we observed a high number of targets associated with iron overload, suggesting inhibition of this process. This finding is interesting taking into account that iron level increases risk of sepsis and severe COVID-19^34^.

Similarly, to the results obtained from our study, the **miR-10b** expression levels were found significantly lower in COVID-19 patients compared to controls. Additionally, the miR-10b expression significantly negatively correlated with the IL-2 and IL-8 serum levels as well as with the age of patients, erythrocyte sedimentation rate and CRP level, suggesting its role in viral infection-induced enhanced cytokine storm^35^. Our results confirmed this relationship showing that miR-10b-5p targets genes significantly associated with IL-2 signalling. An even stronger association was observed for IL-1 regulation of the extracellular matrix. This result points out its potential role of miR-10b-5p the fibrotic process and lung repair^36^. Our enrichment analysis showed that miR-10b-5p strongly regulates COVID-19 targets associated with Myocardial ischemia. Literature data shows that overexpression of miR‐10b‐5p in the murine model of MI significantly reduced MI size, improved cardiac function, and inhibited apoptosis^37^. miR-10b-5p was identified by us as a key regulator of ACE2 network which aligns with COVID-19 cardiovascular effects.

**MiR-302c-5p** role is mainly investigated in viral infections, nervous system diseases and cancers^38,39^. The miR-302 has not yet been described specifically in the context of the SARS-CoV2 infection. Lower expression of miR-302a and increased levels of NF-κB direct target, interferon regulatory factor-5 (IRF-5) was found in patients with influenza A infection compared to controls. IRF-5 promotes viral replication, the opposite effect was shown by miR-302a treatment, which inhibits viral replication^40^. This can be the explanation of our observation as in our study we found that miR-302c expression decreased over time in patients with COVID-19. Moreover, our previous bioinformatic analysis of the ACE2 interaction network showed that miR-302c-5p modulated a high number of virus-linked proteins^1^. Therefore, we suggest that miR-302c-5p might be a prognostic biomarker for COVID-19 patients. Additionally, members of miRNA-302 family members are considered potential biomarkers for the diagnosis of acute heart failure^41^. This aligns with our results regarding the CVDs related ontological terms associated with targets of miR-302c-5p, especially Arterial hypertension.

The enrichment analysis performed in this study focused on COVID-19 related targets of analyzed miRNAs showed that all of them strongly regulated CVDs-related phenotypes. This result is consistent with our in silico findings regarding the role of ACE-2 interaction network in Cardiovascular outcomes in COVID19. That strong regulation of the ACE-2 network can have an effect in developing, or even aggravating, the CVDs phenotypes^42,43^. But also we observed that analyzed miRNAs regulated COVID-19 related genes associated with other phenotypes like Chronic Obstructive Pulmonary Disease (COPD) (miR-10b-5p), Glomerulonephritis (miR-124-3p), Prostate Cancer (miR-200b-3p), Amyotrophic Lateral Sclerosis (miR-26b-5p), and Endometriosis (miR-302c-5p) which could be important factors for consideration of post-COVID19 complications.

We identified the following top targeted pathways affected by analyzed miRNAs: IL-2 signalling pathways, signaling events mediated by T cell protein tyrosine phosphatase (TC-PTP) and HIF-1 transcriptional activity in hypoxia which would require further investigation. Regarding IL-2, certain studies propose that there is an elevation in the IL-2 cytokine family levels during the infection, leading to a heightened inflammatory response and the potential occurrence of a cytokine storm^44^. Elevated cytokine levels worsen prognosis in immunocompromised patients, while in those without underlying conditions, IL-2 family upregulation correlates with disease severity. Conflicting studies exist, with some showing no significant cytokine changes. Healthy individuals post-vaccination exhibit IL-2 upregulation, but immunocompromised patients require booster doses for efficacy. The IL-2 cytokine family holds potential as immunotherapy for COVID-19 thus it seem necessary to validate those findings on protein level.

TC-PTP is an enzyme involved in the regulation of immune responses and cellular signaling, especially in JAK-STAT pathway, important in COVID19 progression^45^. Crucial in this process ORF3a induces renal tubule injuries by activating NF-κB and STAT3 signaling. It promotes STAT3 activation by interacting with the ubiquitin E3 ligase TRIM59. This interaction leads to the dissociation of the phosphatase TC-PTP from STAT3, inhibiting STAT3 dephosphorylation and resulting in prolonged STAT3 activation^46^. Investigation of the role of TC-PTP signalling seems important for better understanding the COVID progression.

As mentioned before, HIF-1α is a key activator for both SARS-CoV-2 infection and inflammatory response and plays a role in aggravation of COVID19^21^. There are significant roles of SARS-CoV-2 ORF3a and HIF-1α in virus infection and pro-inflammatory responses. In one study RNA sequencing reveals dysregulation in HIF-1α signaling, immune response, and metabolism pathways in COVID-19 patients^21^. Clinical analyses highlight increased HIF-1α production, inflammatory responses, and mortality rates in elderly patients. HIF-1α TC-PTP and HIF-1α ORF3a related induces mitochondrial damage and Mito-ROS production, promoting HIF-1α expression, facilitating SARS-CoV-2 infection and cytokine production. Importantly, HIF-1α broadly promotes infection in various viruses. Overall, ORF3a-induced HIF-1α aggravates viral infection and inflammatory responses during SARS-CoV-2 infection, establishing HIF-1α's pivotal role in promoting SARS-CoV-2 infection and inducing pro-inflammatory responses in COVID-19^21,47^. Thus it seem important to evaluate the better the role of this factor in COVID19 progression.

In this study we identified and validated miRNAs which could serve as novel, predictive biomarkers and their targets of the COVID-19 long term hospitalisation, and can be used for early stratification of patients and prediction of severity of infection development in an individual. We found that low delta miR-200b-3p expression presents predictive utility in assessment of the hospital length of stay and/or death. Analysis focused on COVID-19 associated genes showed that all analysed miRNAs are strongly affecting multiple signalling and disease pathways related to CVDs which could be explained by their strong interaction with the ACE2 network. Identifying novel biomarkers and implementing machine learning based tools enabling feature identification and bioinformatics may improve clinical data interpretation and prediction of the outcome in patients with COVID-19.

## Methods

### Study group

Seventy-nine patients diagnosed (positive nasopharyngeal swab PCR test) with COVID-19 were enrolled to the study during the third pandemic wave in Poland (January-May 2021). Patients were admitted to the Military Institute of Medicine in Warsaw^2^. Thirty-two age and sex and cardiovascular risk factors matched SARS-CoV-2 infection-free participants were sampled as a control group in out-patients clinic. Blood samples of COVID-19 patients were collected at three different time points, whereas control group patients were sampled only once^2^.

Medical records were obtained and demographic, clinical and laboratory data were presented in Table 1. Whole blood samples were collected by using Tempus™ Blood RNA Tube (Applied Biosystems) and were kept in -80 ˚C until the day of experiments in Medical University of Warsaw. No freeze–thaw cycles were performed during the experiments.

### RNA preparation, detection, and quantification of miRNAs by Quantitative PCR

We isolated total RNA using Tempus™ Spin RNA Isolation Kit (Invitrogen). Next, we performed a reverse transcription reaction using the TaqMan Advanced miRNA cDNA synthesis kit (ABI, California, USA) following the guidelines provided by the manufacturer. Afterwards, miRNA expressions were detected by quantitative polymerase chain reaction (qPCR) using TaqMan miRNA Assay kits (ABI, California, USA, catalog number A25576, assay ID: 478418_mir; 480901_mir; 478494_mir; 477963_mir; 478800_mir) by using CFX384 Touch Real-Time PCR Detection System (BioRad Inc. Hercules, California, USA). Cel-miR-39 was spiked-in during miRNA extraction phase and was used as an external normalizer. All reactions were performed in triplicates, and mean values were used in statistical analysis as described before^48,49^. MiRNA expressions were expressed as 2-ΔΔCT^50^, results were log-10 transformed for statistical analysis.

### Statistical analysis

Categorical variables were presented as a number and percentage, continuous variables were expressed as mean ± standard deviation (SD) or median and interquartile range (IQR) based on the distribution of the data. They were selected based on the normality of distribution evaluated by Shapiro–Wilk test. Depending on the normality of the distribution, the Student's t-test or Mann–Whitney test was used for unpaired samples and the Wilcoxon test for paired samples. One-way ANOVA or Kruskal–Wallis tests were used to compare more than two groups, accordingly. To assess the predictive value of miR-200b-3p changing over time (delta miR-200b-5p expression) for increased hospital length of stay or death in follow-up as a composite endpoint we used receiver operating characteristic (ROC) analysis. Low delta miR-200b-3p expression (< -0.522 expression), age (years), male sex, BMI > 30, hypertension, diabetes, smoking, and coronary artery disease were included in the multivariate logistic regression analysis model. All tests were two-sided with a significance level of p < 0.05. Calculations were performed using SPSS version 22.0 (IBM Corporation, Chicago, USA). Graphs were improved by Adobe Illustrator 24.0.2.

### Bioinformatic analysis

#### SHAP analysis of the clinical data

We applied SHAP analysis to the cleaned clinical dataset considering the ‘Demographics’ and ‘Laboratory Results’ Section. For this analysis, we considered as output of interest (target) the column ‘death + 21day_stay’, which is a binary feature referring to patients who died or not after 21 days after the hospital admission. Then, we ran the XGBoost classifier ten times to check robustness, which led to similar results with low variation in the top 10 features.

#### Gene lists selection

We downloaded the following lists of genes related to our processes and phenotypes of interest. From the Gene Ontology (GO) database we obtained gene lists associated with coagulation (17 GO terms) platelet activity (26 GO terms), and inflammatory response (22 GO terms) using the biomartR R package^51^ (Supplementary file 2, spreadsheet GO terms). From the Malacards database we obtained gene lists related to thrombosis (https://www.malacards.org/; ID:THR024). From the Disgenet database we obtained multiple gene lists: related to thrombosis (C0040053), Diabetes Mellitus Insulin-Dependent (genes with at least 2 NCBI publications; C0011854), COVID19 (genes with at least 2 NCBI publications; https://www.disgenet.org/covid/genes/summary/), White blood.cell abnormality (C0152009), C reactive protein.measurement (C0201657), Iron Overload (C0282193), Iron deficiency (C0240066), Lymphocyte Count measurement (C0200635) related to clinical data. List of ACE2 related genes was obtained from previous publication regarding ACE2 network in COVID19^1^. D-dimer related gene lists were obtained from literature data^52^. All gene lists are available in Supplementary file 2 (spreadsheet gene lists combined). All gene symbols were unified using the NCBI annotation file.

#### miRNA target prediction and ranking

We used on all steps of bioinformatic analyses we used “wizbionet” R-package https://github.com/wizbionet/wizbionet/blob/master/doc/vignette_wizbionet.md^53^. To identify targets of analyzed miRNAs we used the multiMiR R package^54^. We searched the top 20% hits among all conserved and non-conserved target sites in 14 target prediction databases. To evaluate how many targets from each gene list of interest were regulated by analysed miRNAs we used wizbionet R package^53^.

#### Enrichment analysis

Enrichment analysis was done using the API for EnrichR database by implementing Hypergeometric test with Benjamini and Hochberg correction, while the reference was the human genome. For all statistical analyses, the significance cutoff was set to adjusted p-value ≤ 0.05. We performed enrichment analyses using the following databases: ARCHS4_Tissues, BioPlanet_2019, Elsevier_Pathway_collections, and GWAS_Catalog_2019. Data visualization of the overlap between top five most significant pathways for each analysed miR was performed in R using ggplot2 and ggrepel libraries.

### Ethics approval

This study was performed in line with the principles of the Declaration of Helsinki. Approval was granted by the Ethics Committee of Medical University of Warsaw on 14th October (2020 KB/160/2020).

### Consent to participate

Informed consent was obtained from all individual participants included in the study.”

### Supplementary Information

Supplementary Information 1.

Supplementary Information 2.

## Data Availability

The datasets generated during and/or analysed during the current study are not publicly available due to ongoing analysis but are available from the corresponding author on reasonable request.

## Abbreviations

ACE2: Angiotensin converting enzyme 2
ARDS: Acute respiratory distress syndrome
COVID-19: Coronavirus disease 2019
CV: Cardiovascular
CVD: Cardiovascular diseases
DM: Diabetes melitus
HF: Heart failure
miRNAs: MicroRNAs
ncRNAs: Non-coding RNAs
NT-proBNP: N-terminal pro-B-type natriuretic peptide
SARS-CoV-2: Severe acute respiratory syndrome coronavirus 2
