# Supplementary material for: Alteration of circulating ACE2-network related microRNAs in patients with COVID-19
Source: Sci Rep. 2024 Jun 12;14:13573. doi: 10.1038/s41598-024-58037-3 (PMC11169442; doi:10.1038/s41598-024-58037-3)
Supplement: Supplementary file 1 — Supplementary Information 1. [file 41598_2024_58037_MOESM1_ESM.docx]

**Supplemental Table 1.** Statistical estimates for prediction of increased hospital length of stay and/or death by delta of miR-200b-3p (7-days post admission changing to the day of admission).

| **MiRNA** | **AUC** **(95% CI)** | **p-value** | **Cut-off** | **Sensitivity** | **Specificity** | **PPV** | **NPV** |
| --- | --- | --- | --- | --- | --- | --- | --- |
| Delta miR-200b-3p | 0.730 (0.61-0.86) | 0.002 | -0.522* | 69% | 72% | 60% | 79% |

Abbreviations: AUC, area under the curve; CI, confidence interval; PPV, positive predictive value; NPV, negative predictive value. *Changing of 7-days post admission to the day of admission data was used for the cut-off value.

**
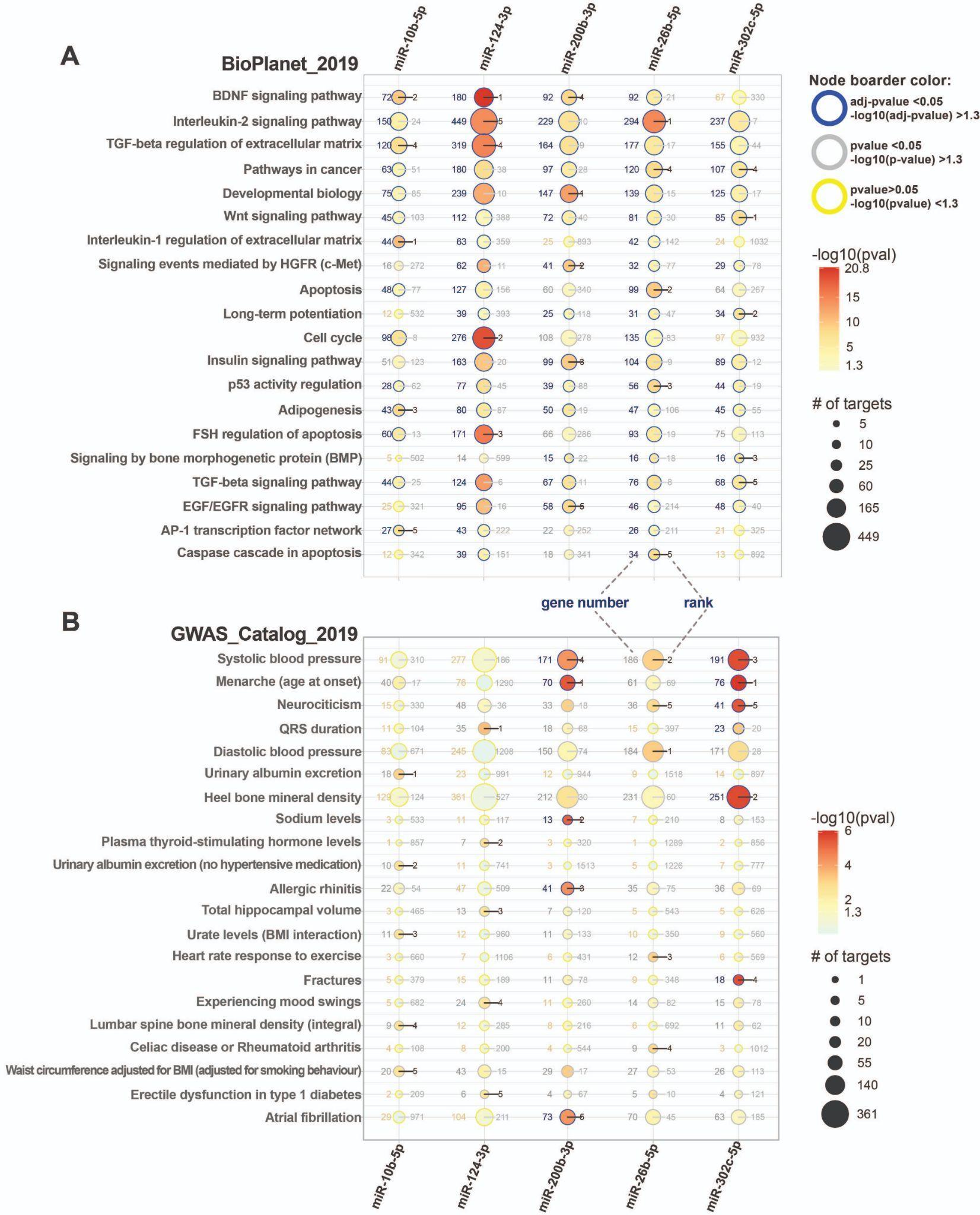
**

**Supplementary Figure 1.** Top 5 significantly enriched pathways (A) and GWAS phenotypes (b) associated with all targets of ACE2 network related miRNAs. Analysis was performed using EnrichR API. The adjusted p-values show categories which are more likely to have biological meanings. The color gradient is related to corresponding adjusted p-values. Blue color indicates high p-values (low enrichment), and red color indicates low p-values (high enrichment). The size of the dots is associated with the number of enriched genes.

## Supplementary file 2 (xlsx) includes the following sheets:

## Supplementary file 2 (xlsx) includes the following sheets:

## Sheet 1 - Gene Ontology terms related to coagulation, inflammation, and platelet activity, used for retrieving gene lists using the biomartr R package.

## Sheet 2 - Combined gene lists used in the study. In column A and P are shared official gene symbols. In columns Q and R Entrez Gene IDs and ENSG ID of analyzed genes. In columns B:N are analyzed gene lists with specified gene number after symbol x. In column O is the number of occurrences on a given gene on analyzed gene lists. The presence of the gene on a gene list is marked as TRUE. The genes were merged using wizbionet lists_combiner R function.

## Sheet 3 - Combined targets (genes) lists for analyzed miRNAs. In column A is presented analyzed target, in columns B:F its association expressed as TRUE/FALSE with analyzed miRNAs (has-miR-10b-5p, hsa-miR-124-3p, hsa-miR-200b-3p, hsa-miR-26b-5p,hsa-miR-302c-5p). In column G is the number of occurrences of given gene as target of analyzed miRNAs.
